# Dysregulated high-density lipoprotein and low-density lipoprotein subfractions increase metabolic dysfunction-associated steatotic liver disease risk: a study of patients across body mass index categories

**DOI:** 10.3389/fnut.2026.1737860

**Published:** 2026-02-13

**Authors:** Hao-Yun Yu, Jia-Qi Zhang, Pei-Qi Sun, Qing-Hua Li, Hong Lin, Jia-Yang Wang, Xiao-Jing Qian, Xiao-Di Yang, Cheng Hu, Ping Tian, Yuan-Ye Jiang, Guo-Qiang Lin

**Affiliations:** 1 Department of Gastroenterology, Putuo Hospital, Shanghai University of Traditional Chinese Medicine, Shanghai, China; 2 State Key Laboratory of Discovery and Utilization of Functional Components in Traditional Chinese Medicine, Innovation Research Institute of Traditional Chinese Medicine Shanghai University of Traditional Chinese Medicine, Shanghai, China; 3 Department of Pharmacy, Shanghai Municipal Hospital of Traditional Chinese Medicine, Shanghai University of Traditional Chinese Medicine, Shanghai, China; 4 Experiment Center for Science and Technology, Shanghai University of Traditional Chinese Medicine, Shanghai, China

**Keywords:** biomarkers, body mass index, lipoprotein subfractions, NMR metabolomics, metabolic dysfunction-associated steatotic liver disease

## Abstract

**Background:**

To investigate biomarker differences among patients with metabolic dysfunction-associated steatotic liver disease (MASLD) across body mass index (BMI) types, we analyzed clinical data from 2,013 subjects and serum samples from 402 patients. The clinical characteristics and lipoprotein subclass profiles were evaluated.

**Methods:**

Participants were grouped based on BMI into overweight MASLD (113 participants), overweight controls (107 participants), lean MASLD (83 participants), and lean controls (99 participants). Serum samples from each group underwent nuclear magnetic resonance-based metabolomic analyses, and clinical and omics data were compared between the lean and overweight MASLD groups and paired control cohorts.

**Results:**

Our study demonstrated distinct omics characteristics for lean MASLD compared with their overweight equivalents. Metabolomic analysis of the serum from the four groups identified six lipoprotein subclasses with significant diagnostic accuracy (area under the curve (AUC) > 0.7), unique to lean individuals with MASLD. In contrast, overweight patients with MASLD had 13 unique lipoprotein subclasses that exhibited a high diagnostic value. These lipoproteins correlate with clinical parameters, such as uric acid, urea, creatinine, eosinophils, blood glucose, and alanine aminotransferase.

**Conclusion:**

Lean and overweight patients with MASLD display unique lipoprotein omics characteristics in an Asian population, primarily involving high-density lipoprotein (HDL) and low-density lipoprotein (LDL) subcomponents, suggesting their potential as effective biomarkers.

## 1 Introduction

MASLD is defined as the excessive accumulation of lipids in the liver in the absence of a history of alcohol-related liver disease. MASLD is one of the most widespread liver diseases globally, affecting nearly 25% of individuals (1, 2). Approximately 10–30% of individuals diagnosed with MASLD advance to metabolic dysfunction-associated steatohepatitis (MASH), a significant factor in cirrhosis and hepatocellular carcinoma (3–5). Currently recognized pathogeneses include obesity, insulin resistance, and inflammatory responses (6, 7).

Because MASLD is intricately linked to metabolic syndrome, it often occurs in individuals with type 2 diabetes and obesity. Previous studies have primarily focused on overweight or obese patients (8–14). However, individuals with a lower body mass index (BMI) (e.g., Asians with a BMI < 23 kg/m^2^) can still develop MASLD, commonly termed as non-obese or lean MASLD, which is defined by a lower overall BMI threshold but increased visceral adipose tissue (15–17). Lean MASLD is most prevalent in Asian populations and most biological studies have been conducted on these individuals (18, 19). Compared to overweight patients with MASLD, lean patients with MASLD demonstrate less severe manifestations of metabolic syndrome; however, some studies indicate that similar or even more severe hepatitis or liver fibrosis may occur (20–23). Additionally, compared to healthy controls, individuals with lean MASLD face heightened risks of dyslipidemia, hypertension, insulin resistance, and diabetes (18, 19, 24). Therefore, lean MASLD is considered a distinct subtype characterized by different metabolic features, pathogeneses, and clinical outcomes (17, 24, 25). However, the association between lean and overweight patients with MASLD remains unclear, and distinct pathophysiological mechanisms require further investigation.

Given the progressive tendencies of MASLD, which may increase the risk of cardiovascular disease and certain malignancies, early diagnosis and interventional therapy are particularly important. The definitive gold standard for diagnosing MASLD remains liver biopsy; however, its implementation is constrained by its invasive nature and risk of complications. Currently, initial screening and evaluation are often performed clinically in conjunction with imaging and noninvasive scoring.

Metabolomics is an emerging histological technology that enables non-invasive detection of diseases. Recent studies on metabolomics in MASLD have focused on small-molecule metabolites and lipidomics (13, 26, 27). Research has identified a relationship between circulating lipoprotein component levels and MASLD (28–32). However, most studies on lipoproteins have focused on routine blood tests and have not delved deeper into their subclass distributions. Compared to conventional detection techniques, nuclear magnetic resonance (NMR) not only provides information on the total class of lipoproteins but also reveals the detailed distribution characteristics of lipoprotein subclasses, including size, density, and particle number, allowing for a more detailed classification, which is not possible with mass spectrometry platforms (33).

However, the relationship between lipoprotein subfractions and MASLD has not been adequately investigated. Therefore, this study delves into the association between altered lipoprotein profiles and MASLD using clinical features and NMR metabolomics, with the aim of providing novel insights and approaches for the prevention, diagnosis, and management of the condition.

## 2 Results

### 2.1 Clinical and biochemical characteristics of subjects

**Figure 1:**
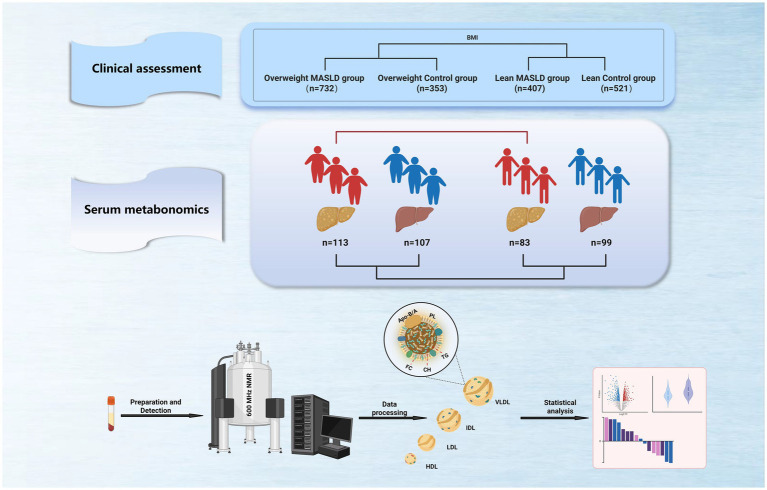
Overview of study design and lipoprotein metabonomics. A. Using a BMI of 23 as the cutoff value, the subjects were divided into four groups: overweight MASLD group, overweight control group, lean MASLD group, and lean control group. (For detailed inclusion and exclusion criteria for participants, see the ‘study subjects’ section of the text.)The clinical data were collected from a cohort of 2013 participants. B. Samples from a subset of participants in each group were selected for serum metabolomics analysis. C. Serum samples were analyzed using NMR-based metabolomics techniques, including the detection of lipoproteins and metabolites, data extraction, and subsequent statistical analysis.

We collected clinical data from 2013 subjects, divided into 4 groups based on BMI and disease status, and randomly selected proportionally balanced samples from each group for serum metabolomics analysis. Figure 1 shows the overall study design. Tables 1–6 present the demographic, clinical, and biochemical characteristics of the subjects. Our results revealed a significant increase in the incidence of MASLD among middle-aged populations, with a higher prevalence in this age group (Table 1). Among overweight patients with MASLD, 50.3% were male (546 individuals) and 49.7% were female (539 individuals), whereas in non-overweight patients with MASLD, the proportion of females was as high as 73.1% (Table 1). Compared to their respective healthy control groups, both non-overweight and overweight patients with MASLD exhibited a slightly increased BMI. Although the average levels of transaminases in non-overweight patients with MASLD were within the normal range, both non-overweight and overweight patients with MASLD demonstrated elevated levels of alanine aminotransferase (ALT), aspartate aminotransferase (AST), alkaline phosphatase (AKP), *γ*-glutamyltransferase (γ-GT), and cholinesterase (CHE) compared with healthy controls (Table 3).

**Table 1:** Demographic data and physical characteristics of the study population.

Variables	Overweight MASLD (*n* = 732)	Overweight control (*n* = 353)	Lean MASLD (*n* = 407)	Lean control (*n* = 521)	*P*		
					OM-OC	LM-LC	OM-LM
Demographic data & physical characteristics							
Gender (M/F)	392/340	154/199	135/272	115/406	<0.001	<0.001	<0.001
AGE (year)	62.00 (51.00,69.00)	53.50 (41.00,63.75)	50.00 (37.50,63.75)	44.00 (29.00,57.00)	<0.001	<0.001	<0.001
BMI (kg/m^2^)	25.05 (23.94,26.61)	24.78 (23.81,25.94)	21.7 (20.72,22.53)	20.50 (19.24,21.45)	<0.001	<0.001	<0.001
Height (cm)	160.00 (156.00,163.75)	159.00 (155.00,165.00)	161.00 (158.00,166.00)	160.00 (157.00,165.00)	1.000	1.000	0.023
Weight (kg)	64.00 (60.00,68.00)	63.50 (58.00,68.00)	56.00 (53.00,59.00)	53.00 (48.00,56.00)	0.007	<0.001	<0.001
FIB4 index	1.88 (1.37,2.51)	1.17 (0.85,1.59)	1.57 (1.10,2.19)	1.59 (1.06,2.25)	<0.001	0.034	<0.001

Continuous variables are shown as mean standard deviation or median [25^th^, 75^th^] percentile and were analyzed using the Shapiro–Wilk test or the Kruskal–Wallis H test, and multiple comparisons of biased information were performed using the Bonferroni method.

**Table 2:** Blood routine examination of the study population.

Variables	Overweight MASLD (*n* = 732)	Overweight control (*n* = 353)	Lean MASLD (*n* = 407)	Lean control (*n* = 521)	*P*		
					OM-OC	LM-LC	OM-LM
Blood routine examination							
FBG (mmol/L)	5.20 (4.80, 6.15)	5.00 (4.60,5.40)	5.10 (4.70,5.70)	4.80 (4.50,5.20)	<0.001	<0.001	<0.001
WBC (×10^9^/L)	5.60 (4.80,6.50)	5.40 (4.83,6.58)	5.40 (4.83,6.20)	4.90 (4.30,5.70)	0.139	0.001	0.305
RBC (%)	4.44 (4.23,4.66)	4.41 (4.14,4.61)	4.39 (4.11,4.70)	4.15 (3.86,4.43)	0.014	<0.001	0.004
HGB (g/L)	133.50 (127.00,141.75)	132.50 (124.00,141.00)	133.00 (123.25,140.00)	127.50 (120.00,134.03)	0.008	<0.001	<0.001
HCT (%)	40.45 (38.30,41.80)	39.80 (37.50,41.90)	39.65 (37.28,41.80)	37.45 (35.28,39.60)	0.003	<0.001	<0.001
MCV (fL)	90.85 (88.23,93.35)	90.75 (87.55,92.85)	89.55 (87.33,92.08)	91.05 (87.80,93.18)	1.000	1.000	0.001
MCH (pg)	30.5 (29.43,31.50)	30.70 (29.50,31.50)	30.15 (29.20,31.28)	31.05 (29.70,32.18)	1.000	1.000	<0.001
MCHC (g/L)	334.00 (328.25,340.00)	336.00 (330.00,341.00)	336.00 (329.25,341.00)	339.00 (331.00,346.00)	0.605	1.000	0.942
PLT (×10^9^/L)	184.50 (159.25,198.00)	205.50 (181.25,224.00)	201.50 (179.25,213.50)	180.50 (158.25,195.75)	1.000	1.000	<0.001
RDW (×10^12^/L)	12.50 (12.20,13.20)	12.55 (12.00,13.20)	12.30 (11.90,13.20)	12.60 (11.90,13.38)	0.112	0.652	0.560
MPV (fL)	11.15 (10.03,11.88)	10.90 (10.00,11.50)	11.00 (10.40,11.60)	10.90 (10.01,12)0.00	0.070	0.944	0.594
Neutrophil (%)	56.60 (50.30,62.13)	57.00 (50.18,63.45)	56.20 (50.28,62.88)	53.35 (47.94,61.88)	0.766	0.064	0.796
Lymphocyte (%)	33.90 (27.90,40.80)	32.15 (27.63,39.20)	34.85 (27.03,39.98)	36.23 (26.35,43.15)	1.000	0.797	1.000
Monocyte (%)	6.85 (5.73,7.80)	7.00 (6.13,8.50)	6.50 (5.83,7.60)	7.20 (6.23,8.48)	0.132	1.000	0.331
Eosinophil (%)	1.40 (0.93,2.20)	1.60 (1.1,2.55)	1.50 (0.90,2.70)	1.60 (0.80,2.80)	1.000	1.000	0.019
Basophils (%)	0.40 (0.30,0.60)	0.54 (0.31,0.70)	0.40 (0.30,0.60)	0.48 (0.30,0.70)	0.208	0.049	0.143

Continuous variables are shown as mean standard deviation or median [25th, 75th] percentile and were analyzed using the Shapiro–Wilk test or the Kruskal–Wallis H test, and multiple comparisons of biased information were performed using the Bonferroni method.

**Table 3:** Renal and liver function tests of the study population.

Variables	Overweight MASLD (*n* = 732)	Overweight control (*n* = 353)	Lean MASLD (*n* = 407)	Lean control (*n* = 521)	*P*		
					OM-OC	LM-LC	OM-LM
Renal function tests							
BUN (mmol/L)	5.50 (4.60,6.40)	5.05 (4.10,6.18)	5.05 (4.30,5.95)	4.70 (4.03,6.00)	0.227	0.023	0.123
Cr (μmol/L)	61.50 (53.00,68.75)	61.00 (53.00,69.00)	60.00 (52.25,67.00)	57.50 (53.00,62.75)	1.000	0.085	<0.001
UA (μmol/L)	341.50 (292.25,396.75)	294.00 (268.25,345.00)	297.50 (255.25,354.75)	260.50 (222.50,297.50)	<0.001	<0.001	<0.001
GFR (mL/min)	95.20 (82.23,112.10)	101.98 (85.76,117.13)	109.43 (90.78,122.93)	107.85 (91.69,130.87)	0.036	0.012	<0.001
Liver function tests							
TB (μmol/L)	13.31 (9.06,17.39)	12.90 (9.00,17.00)	13.00 (9.00,17.10)	13.64 (9.61,18.29)	0.771	0.169	0.966
DB (μmol/L)	2.35 (1.53,3.59)	2.40 (1.53,3.29)	2.50 (1.66,3.47)	2.83 (1.70,4.38)	0.281	0.700	0.974
AKP (U/L)	76.50 (63.42,93.35)	71.64 (54.44,87.75)	70.00 (54.00,83.21)	62.00 (47.60,79.72)	<0.001	<0.001	<0.001
TP (g/L)	73.00 (71.00,76.00)	71.11 (68.48,74.00)	74.00 (69.03,76.00)	72.00 (68.20,75.00)	<0.001	0.407	1.000
ALB (g/L)	42.93 (38.54,46.69)	43.46 (38.25,46.18)	42.00 (35.92,46.28)	42.00 (37.76,45.66)	0.389	1.000	0.120
γ-GT (U/L)	26.00 (17.25,58.32)	22.00 (15,43.75)	22.00 (14,53.09)	20.00 (12.25,125.71)	<0.001	0.702	<0.001
CHE (U/L)	8735.05 (7412.96,9919.23)	8081.5 (6574.06,9529.95)	8499.67 (7255.42,9683.32)	7462.99 (5934.85,8913.82)	<0.001	<0.001	0.024
ALT (U/L)	14.00 (11.25,20.75)	15.00 (11.00,25.00)	12.50 (8.80,18.14)	9.00 (7.00,14.00)	0.014	<0.001	<0.001
AST (U/L)	23.56 (19.00,28.44)	19.00 (12.09,25.00)	21.50 (17.25,26.50)	19.64 (17.00,25.26)	<0.001	<0.001	0.001
TBA (μmol/L)	5.82 (3.00,8.77)	4.74 (2.00,7.98)	4.00 (2.00,10.54)	4.00 (2.00,9.38)	0.323	0.281	0.111

Continuous variables are shown as mean standard deviation or median [25th, 75th] percentile and were analyzed using the Shapiro–Wilk test or the Kruskal–Wallis H test, and multiple comparisons of biased information were performed using the Bonferroni method.

**Table 4:** Lipid panel of the study population.

Variables	Overweight MASLD (*n* = 732)	Overweight control (*n* = 353)	Lean MASLD (*n* = 407)	Lean control (*n* = 521)	*P*		
					OM-OC	LM-LC	OM-LM
Lipid panel							
HDL-C (mmol/L)	1.26 (1.14,1.42)	1.31 (1.08,1.49)	1.36 (1.17,1.52)	1.46 (1.29,1.65)	<0.001	<0.001	<0.001
LDL-C (mmol/L)	3.50 (2.93,3.85)	3.45 (2.77,3.99)	3.30 (2.72,3.98)	3.00 (2.43,3.40)	0.016	<0.001	1.000
TC (mmol/L)	5.32 (4.59,5.92)	5.16 (4.33,6.01)	5.09 (4.47,6.16)	4.65 (4.07,5.51)	<0.001	<0.001	1.000
TG (mmol/L)	1.41 (1.06,1.87)	1.19 (0.84,1.65)	1.21 (0.82,1.81)	0.94 (0.68,1.40)	<0.001	<0.001	<0.001
APOA1 (g/L)	1.40 (1.21,1.73)	1.40 (1.23,1.68)	1.49 (1.31,1.78)	1.61 (1.34,2.05)	0.742	0.004	0.001
APOB (g/L)	1.04 (0.84,1.75)	1.03 (0.80,1.80)	0.94 (0.74,1.28)	0.91 (0.70,2.75)	0.241	0.852	1.000
Lp(a) (mg/L)	80.00 (40.33,218.38)	126.99 (54.25,257.92)	73.00 (45,15.12)	87.79 (42.00,234.25)	0.001	0.340	1.000

Continuous variables are shown as mean standard deviation or median [25th, 75th] percentile and were analyzed using the Shapiro–Wilk test or the Kruskal–Wallis H test, and multiple comparisons of biased information were performed using the Bonferroni method.

**Table 5:** Thyroid function tests of the study population.

Variables	Overweight MASLD (*n* = 732)	Overweight control (*n* = 353)	Lean MASLD (*n* = 407)	Lean control (*n* = 521)	*P*		
					OM-OC	LM-LC	OM-LM
Thyroid function tests							
TgAb (IU/mL)	132.78 (45.09,285.68)	160.81 (24.87,330.33)	87.83 (11.10,205.55)	140.11 (44.14,328.38)	0.863	0.015	0.188
TPOAb (IU/mL)	67.20 (19.02,128.16)	66.31 (7.39,145.92)	47.36 (1.48,145.38)	72.34 (17.20,148.57)	0.044	0.502	0.183
Tg (ng/mL)	7.02 (3.973,12.60)	8.01 (4.76,14.18)	7.81 (4.73,13.70)	7.29 (3.45,12.92)	0.421	0.910	0.822
TRAb (IU/mL)	0.59 (0.12,1.36)	0.85 (0.39,1.70)	0.52 (0.10,1.23)	0.87 (0.24,2.58)	<0.001	0.003	1.000
T3 (ng/mL)	1.87 (1.49,2.66)	1.74 (1.50,2.23)	1.97 (1.61,2.39)	1.82 (1.41,2.30)	0.003	0.004	1.000
T4 (ng/mL)	101.00 (87.25,115.25)	102.12 (87.72,115.00)	102.00 (92.05,115.26)	98.48 (86.26,108.87)	1.000	0.014	1.000
FT3 (pmol/L)	4.99 (4.23,6.16)	5.01 (4.38,5.72)	5.10 (4.46,6.18)	4.80 (4.16,6.34)	0.601	0.280	0.638
FT4 (pmol/L)	11.13 (9.70,15.44)	12.00 (8.64,15.80)	11.10 (8.83,13.22)	11.06 (8.39,14.19)	0.235	0.085	0.980
TSH (μIU/L)	3.91 (2.08,10.16)	3.61 (1.77,8.37)	3.43 (2.01,8.08)	6.31 (3.07,13.62)	1.000	0.009	1.000

Continuous variables are shown as mean standard deviation or median [25th, 75th] percentile and were analyzed using the Shapiro–Wilk test or the Kruskal–Wallis H test, and multiple comparisons of biased information were performed using the Bonferroni method.

**Table 6:** Tumor marker tests of the study population.

Variables	Overweight MASLD (*n* = 732)	Overweight control (*n* = 353)	Lean MASLD (*n* = 407)	Lean control (*n* = 521)	*P*		
					OM-OC	LM-LC	OM-LM
Tumor marker tests							
CA-242 (U/mL)	5.67 (2.99,13.87)	10.59 (4.23,30.83)	6.36 (3.31,13.15)	9.81 (5.36,27.14)	<0.001	<0.001	1.000
CA50 (U/mL)	5.44 (2.76,10.02)	5.87 (2.62,16.52)	4.66 (2.65,9.09)	5.15 (2.90,11.94)	0.221	0.109	0.176
CEA (ng/mL)	1.67 (1.11,2.66)	1.77 (1.19,3.18)	1.50 (0.97,2.35)	1.55 (1.07,2.60)	0.204	1.000	0.001
AFP (ng/mL)	3.86 (2.83,5.36)	4.07 (2.88,5.89)	3.53 (2.86,4.84)	3.66 (2.81,6.57)	0.185	0.521	0.316
CA19-9 (U/mL)	8.48 (5.01,17.51)	8.20 (3.70,26.51)	9.27 (5.09,16.13)	8.64 (4.77,27.75)	0.330	0.240	0.801
CA72-4 (U/mL)	2.45 (1.00,6.66)	2.65 (1.07,8.35)	1.47 (1.00,4.82)	3.82 (1.46,8.66)	0.033	0.009	1.000
CA211 (U/mL)	1.95 (1.35,2.74)	2.06 (1.59,2.83)	1.96 (1.33,2.49)	1.92 (1.34,2.51)	1.000	0.498	0.491
CA125 (U/mL)	10.18 (7.22,13.76)	11.48 (7.41,18.17)	10.59 (7.17,14.72)	11.57 (7.47,16.08)	0.579	1.000	0.021
NSE (ng/mL)	12.54 (9.25,16.25)	11.73 (9.79,15.14)	12.80 (10.21,16.28)	10.82 (7.97,13.28)	<0.001	<0.001	1.000
SCC (ng/mL)	0.45 (0.31,0.68)	0.50 (0.37,0.90)	0.50 (0.37,0.66)	0.44 (0.30,0.70)	0.935	0.942	0.100

Continuous variables are shown as mean standard deviation or median [25th, 75th] percentile and were analyzed using the Shapiro–Wilk test or the Kruskal–Wallis H test, and multiple comparisons of biased information were performed using the Bonferroni method.

Complete blood count analysis showed that both the lean and overweight MASLD groups had significantly elevated levels of hemoglobin (HGB), hematocrit (HCT), red blood cell count (RBC), and white blood cell count (WBC) (Table 2). Overweight patients with MASLD showed the most significant increases in these indicators. Additionally, individuals with MASLD exhibited markedly elevated fasting blood glucose concentrations compared with healthy controls, suggesting a poorer metabolic status. Although platelet counts were markedly elevated in overweight MASLD patients compared with healthy controls, the differences between the lean and overweight MASLD groups and lean healthy controls were not statistically significant.

Lipid profile analysis (Table 4) revealed significant differences in high-density lipoprotein cholesterol (HDL-C), low-density lipoprotein cholesterol (LDL-C), triglyceride (TG), and apolipoprotein A1 (APOA1) levels among the four groups. This study excluded patients with hyperlipidemia and hypertriglyceridemia; however, both the overweight and lean disease groups exhibited substantially elevated LDL-C and TG concentrations compared with the healthy control group, while HDL-C and APOA1 levels were relatively lower. Furthermore, although total cholesterol (TC) and apolipoprotein B (APOB) levels in both the lean and overweight MASLD groups were elevated compared with their respective healthy control counterparts, these differences were not statistically significant.

A substantial number of observational studies have shown that the incidence of chronic kidney disease (CKD) is markedly higher in patients with MASLD than in those without the condition (34, 35). In contrast to matched healthy controls, individuals with lean MASLD demonstrated substantially heightened concentrations of uric acid (UA) and creatinine (Cr), but only UA concentrations were notably increased in overweight patients with MASLD (Table 3). Several studies have reported that creatinine is a contributing factor to MASLD (36, 37). Our study also observed statistically meaningful variations in creatinine levels between overweight and lean cohorts of patients with MASLD.

### 2.2 Serum metabolomics analysis

**Figure 2:**
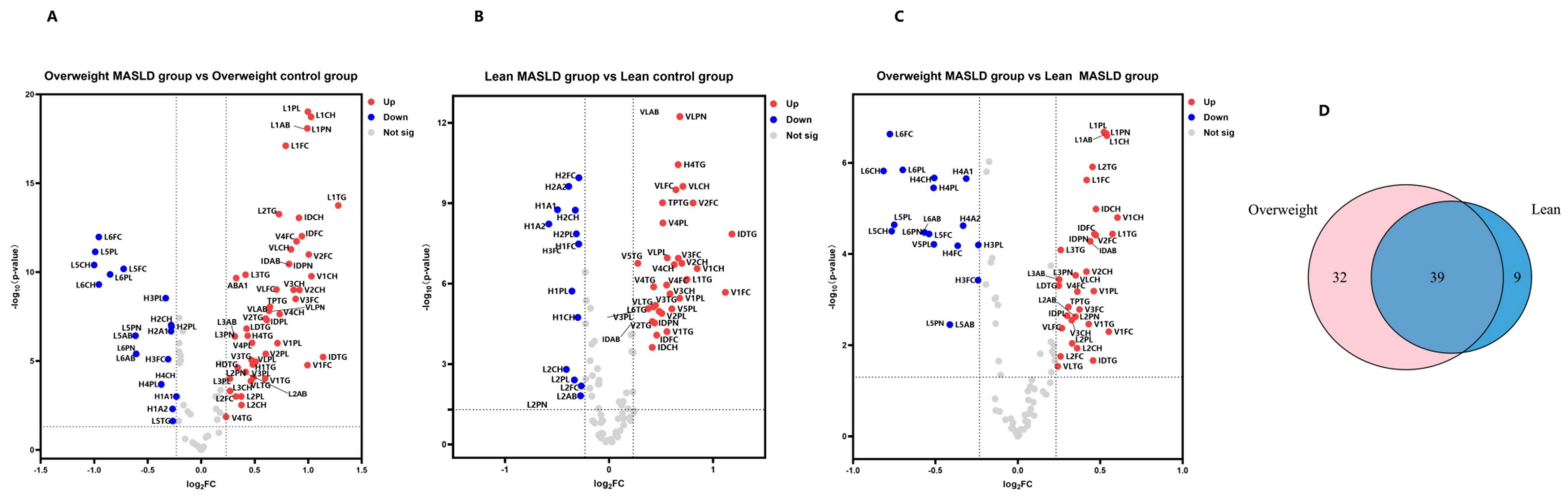
Lipoprotein subfractions differences between lean and overweight MASLD groups. **(A)** Volcano plots of lipoprotein subfractions comparing the overweight MASLD group with the overweight control group. Red circles indicate upregulation in the overweight MASLD group, and blue indicates downregulation (cut-off value of FC > 1.2 or FC < 0.85; *P* (*t*-test) < 0.05). **(B)** Volcano plots of lipoprotein subfractions comparing the lean MASLD cohort and the lean control cohor. **(C)** Volcano plots of lipoprotein subfractions comparing the overweight MASLD group with the lean MASLD group. **(D)** Venn diagram representing the significant commonality of differentially expressed lipoproteins in the lean and overweight groups.

A total of 110 lipoprotein subfraction concentrations and 31 metabolite concentrations were quantified using NMR and data filtering. Lipoprotein subclasses and metabolites with a fold change (FC) > 1.2 or FC < 0.85 (*p* < 0.05) between any two groups were considered differential markers (Tables 7–13). Differentially abundant substances were identified across the BMI groups between the MASLD and control groups. Nine unique lipoprotein subclasses were identified in the lean MASLD group, 32 in the overweight MASLD group, and 39 shared differential lipoproteins between the two groups. (Figure 2D). Specific details are provided in Tables 7–13. Specifically, Tables 7–9 summarize the lipoprotein subclasses unique to the lean and overweight groups, respectively. Tables 10–13 list the lipoprotein subclasses common to both groups, presenting their fold-change (FC) values and direction of change in the disease groups.

**Table 7:** Unique lipoprotein subclasses in overweight MASLD vs. overweight control and lean MASLD vs. lean control.

OM vs. OC			LM vs. LC		
Lipoprotein subclass	FC	Variation	Lipoprotein subclass	FC	Variation
ABA1	1.255	↑	V5TG	1.211	↑
L1PN	1.989	↑	V5PL	1.520	↑
L3PN	1.243	↑	L6TG	1.299	↑
L5PN	0.653	↓	H1CH	0.812	↓
L6PN	0.656	↓	H1FC	0.816	↓
LDTG	1.341	↑	H2FC	0.818	↓
HDTG	1.267	↑	H1PL	0.781	↓
IDPL	1.530	↑	H1A1	0.709	↓
L2TG	1.654	↑	H2A2	0.764	↓
L3TG	1.333	↑	—	—	—

↑ Overrepresentation of the lipoprotein subclass in the serum of patients with MASLD; ↓ underrepresentation of the lipoprotein subclass in the serum of patients with MASLD.

**Table 8:** Unique lipoprotein subclasses in overweight MASLD vs. overweight control and lean MASLD vs. lean control.

OM vs. OC			LM vs. LC		
Lipoprotein subclass	FC	Variation	Lipoprotein subclass	FC	Variation
L5TG	0.832	↓	—	—	—
L1CH	2.037	↑	—	—	—
L3CH	1.205	↑	—	—	—
L5CH	0.500	↓	—	—	—
L6CH	0.515	↓	—	—	—
L1FC	1.728	↑	—	—	—
L5FC	0.605	↓	—	—	—
L6FC	0.515	↓	—	—	—
L1PL	1.996	↑	—	—	—
L3PL	1.205	↑	—	—	—

↑ Overrepresentation of the lipoprotein subclass in the serum of patients with MASLD; ↓ underrepresentation of the lipoprotein subclass in the serum of patients with MASLD.

**Table 9:** Unique lipoprotein subclasses in overweight MASLD vs. overweight control and lean MASLD vs. lean control.

OM vs. OC			LM vs. LC		
Lipoprotein subclass	FC	Variation	Lipoprotein subclass	FC	Variation
L5PL	0.503	↓	—	—	—
L6PL	0.554	↓	—	—	—
L1AB	1.989	↑	—	—	—
L3AB	1.243	↑	—	—	—
L5AB	0.653	↓	—	—	—
L6AB	0.656	↓	—	—	—
H1TG	1.398	↑	—	—	—
H4CH	0.772	↓	—	—	—
H3PL	0.795	↓	—	—	—
H4PL	0.772	↓	—	—	—
H2A1	0.822	↓	—	—	—
H4A2	0.851	↓	—	—	—

↑ Overrepresentation of the lipoprotein subclass in the serum of patients with MASLD; ↓ underrepresentation of the lipoprotein subclass in the serum of patients with MASLD.

**Table 10:** Common lipoprotein subclasses in lean MASLD vs. lean controls and overweight MASLD vs. overweight controls.

Lipoprotein subclass	OM vs. OC		LM vs. LC	
	FC	Variation	FC	Variation
TPTG	1.560	↑	1.428	↑
VLPN	1.551	↑	1.603	↑
IDPN	1.766	↑	1.354	↑
L2PN	1.334	↑	0.827	↓
VLTG	1.380	↑	1.345	↑
IDTG	2.201	↑	2.267	↑
VLCH	1.789	↑	1.636	↑
IDCH	1.884	↑	1.333	↑
VLFC	1.629	↑	1.561	↑
IDFC	1.919	↑	1.374	↑

↑ Overrepresentation of the lipoprotein subclass in the serum of patients with MASLD; ↓ underrepresentation of the lipoprotein subclass in the serum of patients with MASLD.

**Table 11:** Common lipoprotein subclasses in lean MASLD vs. lean controls and overweight MASLD vs. overweight controls.

Lipoprotein subclass	OM vs. OC		LM vs. LC	
	FC	Variation	FC	Variation
VLPL	1.423	↑	1.473	↑
VLAB	1.551	↑	1.603	↑
IDAB	1.766	↑	1.354	↑
V1TG	1.514	↑	1.470	↑
V2TG	1.519	↑	1.334	↑
V3TG	1.386	↑	1.362	↑
V4TG	1.175	↑	1.346	↑
V1CH	2.042	↑	1.799	↑
V2CH	1.890	↑	1.623	↑
V3CH	1.820	↑	1.501	↑

↑ Overrepresentation of the lipoprotein subclass in the serum of patients with MASLD; ↓ underrepresentation of the lipoprotein subclass in the serum of patients with MASLD.

**Table 12:** Common lipoprotein subclasses in lean MASLD vs. lean controls and overweight MASLD vs. overweight controls.

Lipoprotein subclass	OM vs. OC		LM vs. LC	
	FC	Variation	FC	Variation
V4CH	1.664	↑	1.544	↑
V1FC	1.990	↑	2.170	↑
V2FC	2.007	↑	1.750	↑
V3FC	1.844	↑	1.587	↑
V4FC	1.854	↑	1.468	↑
V1PL	1.639	↑	1.600	↑
V2PL	1.519	↑	1.421	↑
V3PL	1.402	↑	1.397	↑
V4PL	1.390	↑	1.432	↑
L1TG	2.429	↑	1.680	↑

↑ Overrepresentation of the lipoprotein subclass in the serum of patients with MASLD; ↓ underrepresentation of the lipoprotein subclass in the serum of patients with MASLD.

**Table 13:** Common lipoprotein subclasses in lean MASLD vs. lean controls and overweight MASLD vs. overweight controls.

Lipoprotein subclass	OM vs. OC		LM vs. LC	
	FC	Variation	FC	Variation
L2CH	1.298	↑	0.752	↓
L2FC	1.252	↑	0.831	↓
L2PL	1.296	↑	0.793	↓
L2AB	1.334	↑	0.827	↓
H4TG	1.352	↑	1.585	↑
H2CH	0.823	↓	0.798	↓
H3FC	0.807	↓	0.817	↓
H2PL	0.825	↓	0.804	↓
H1A2	0.830	↓	0.669	↓

↑ Overrepresentation of the lipoprotein subclass in the serum of patients with MASLD; ↓ underrepresentation of the lipoprotein subclass in the serum of patients with MASLD.

We first compared the lipoproteins and metabolites between the lean MASLD and lean control groups, identifying 48 lipoproteins (33 upregulated and 15 downregulated in the lean MASLD group) (Figure 2B) and eight metabolites with significant differences (Supplementary Figure 2B). The lipoproteins that were elevated in the lean MASLD group primarily consisted of the VLDL and IDL subclasses, whereas the downregulated lipoprotein subclasses were mainly HDL and LDL-2. Changes in lipoprotein profiles between the MASLD and healthy groups are shown in a heat map (Supplementary Figure 1A).

When comparing lipoprotein subclasses and metabolites between the overweight MASLD and overweight control groups, 71 differential lipoproteins (Figure 2A) and 18 differential metabolites (Supplementary Figure 2A) were identified. Among these, 51 upregulated lipoproteins were predominantly associated with LDL, VLDL, and IDL subclasses, whereas 20 downregulated lipoproteins were primarily linked to HDL and smaller, denser LDL particles. Changes in lipoprotein profiles are illustrated in Supplementary Figure 1B.

To explore the biological differences between lean and overweight patients with MASLD, statistical analyses were conducted for the two groups. According to the volcano plot results, 53 lipoproteins (35 downregulated and 18 upregulated in lean MASLD) (Figure 2C) and six differential metabolites (Supplementary Figure 2C) were identified, indicating that lean MASLD represents a distinct pathological condition compared to overweight MASLD. Additionally, cluster heatmap analysis based on FC > 1.2 or FC < 0.85, effectively differentiated the two groups (Supplementary Figure 1C).

### 2.3 Random forest analysis

**Figure 3:**
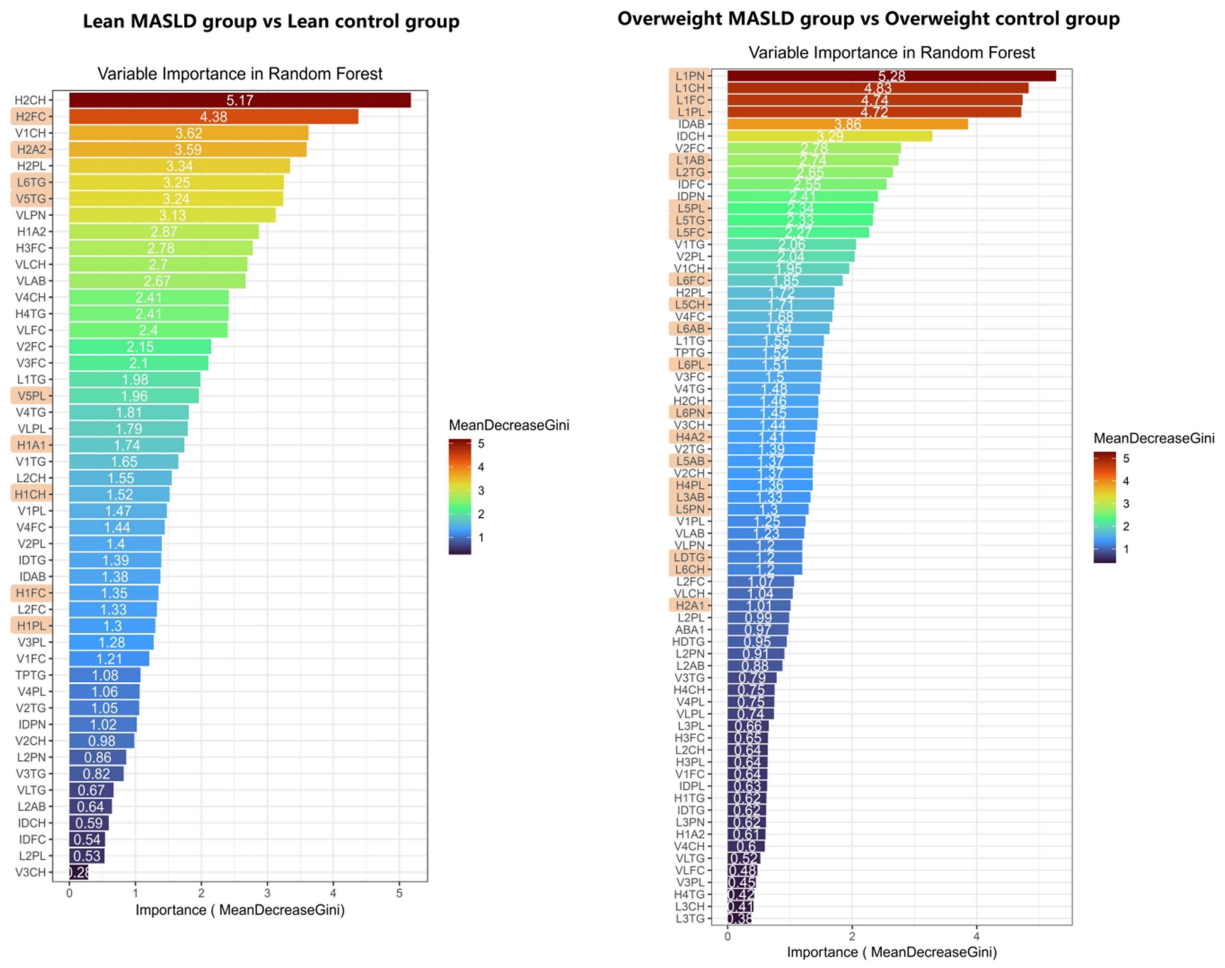
Random forest variable importance plot for lean MASLD group vs. lean control group and overweight MASLD group vs. overweight control group.

To assess the contribution of significantly different lipoproteins to the subgroups, we performed a random forest analysis of the lipoprotein subclasses across the three groups. The contributions of lipoprotein subclasses are illustrated in an importance plot (Figure 3). The model scores were based on the mean decrease in the Gini impurity, with a higher mean value indicating a greater contribution of each feature to the model’s classification accuracy. To evaluate the robustness of the model during the random forest feature selection process and to mitigate the risk of overfitting, we utilized the algorithm’s built-in out-of-bag (OOB) error estimation as an internal validation method. The final model achieved OOB-estimated accuracies of 76.92% (error rate 23.08%) for the lean MASLD group versus the lean control group and 79.01% (error rate 20.99%) for the overweight MASLD group versus the overweight control group (Supplementary Table 1; Supplementary Figure 9). These results demonstrate that, even without introducing an external independent cohort, the model exhibited acceptable generalizability through its intrinsic validation mechanism during the training process.

In the analysis distinguishing the lean MASLD group from the control group, 39 lipoprotein parameters had a contribution score > 1. Among these, nine differential lipoproteins were unique to the lean group, primarily H1A1, H1PL, H1FC, H1CH, H2FC, and H2A2 from the HDL subclasses, V5TG and V5PL from the VLDL subclasses, and L6TG from the LDL subclasses.

In the analysis comparing the overweight MASLD group to the overweight control group, 45 lipoprotein parameters had a contribution score > 1, encompassing the LDL, VLDL, HDL, and IDL subclasses. Among these, 22 differential biomarkers were unique to the overweight group and primarily consisted of LDL subclasses, including LDTG, L1PN, L1CH, L1FC, L1PL, L1AB, L2TG, L3AB, L5PL, L5TG, L5FC, L5CH, L5AB, L5PN, L6FC, L6AB, L6PL, L6PN, and L6CH. HDL subclasses included H4A2, H4PL, and H2A1.

### 2.4 Evaluation of differential metabolite diagnostic efficacy

**Figure 4:**
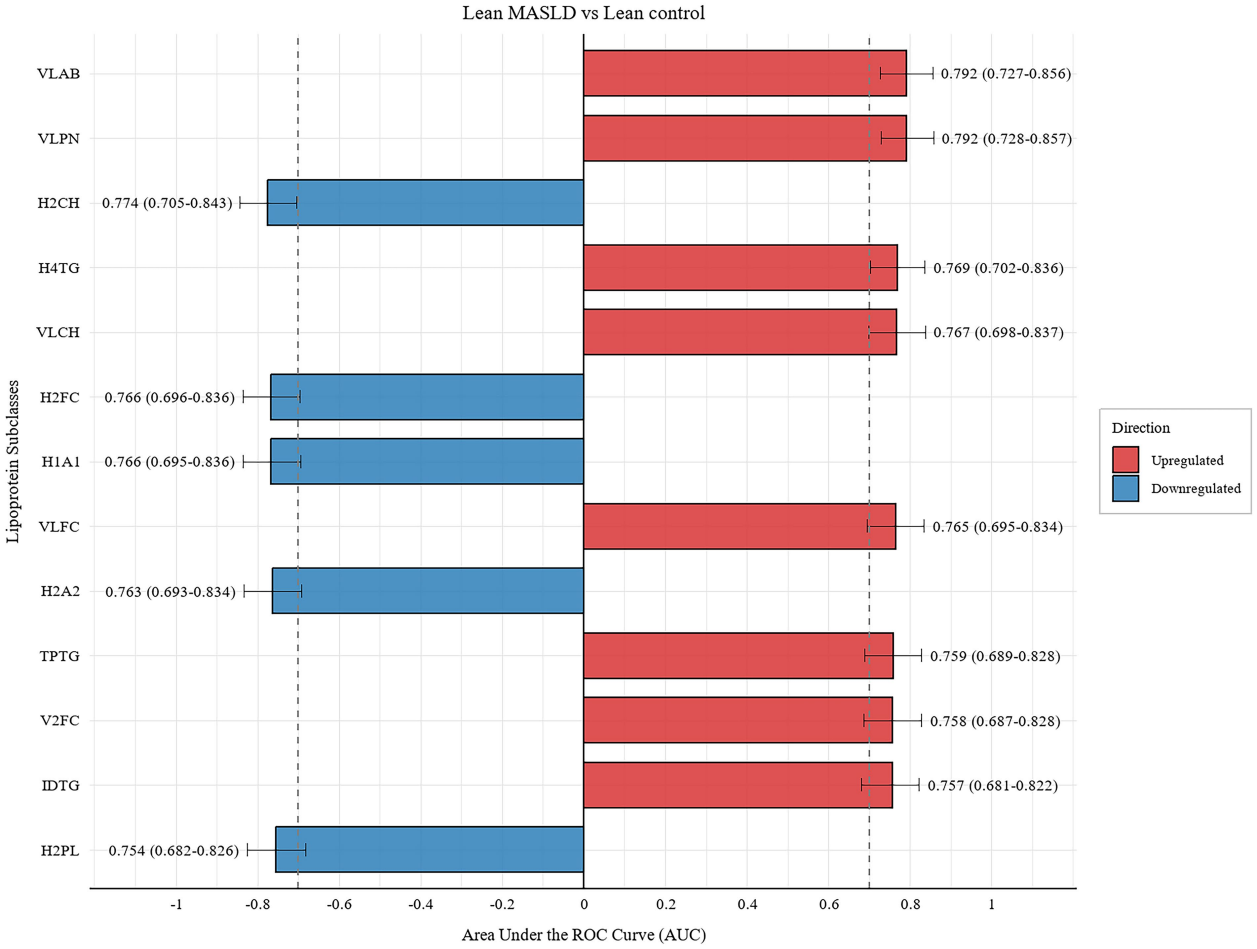
Diagnostic performance of candidate biomarkers with AUC > 0.75 in the lean MASLD group vs. the lean control group. Bar length represents the area under the curve (AUC) for each candidate lipoprotein subclass, with error bars indicating the 95% confidence interval. Lipoproteins highlighted in red and blue denote up- and down-regulated subclasses, respectively.

We conducted receiver operating characteristic (ROC) curve analysis of the clinical lipid testing parameters and found a low diagnostic rate (Supplementary Figure 6). To identify clinically relevant biomarkers, we performed ROC analysis on differential lipoprotein subclasses using an AUC > 0.7, to evaluate the diagnostic capability of candidate biomarkers, ultimately identifying 29 lipoproteins that met the criteria. Figure 4 depicts lipoprotein subclasses with an AUC > 0.75. The remaining subclasses are shown in Supplementary Figure 8. Violin plots of lipoprotein subclasses are shown in Supplementary Figure 3. In order to verify the stability of these 29 lipoprotein subclasses, we conducted Bootstrap analysis, and the results showed that they had high stability (Supplementary Figure 10). In the context of overweight MASLD, lipoprotein V5TG, which is specific to lean MASLD, was elevated in the lean MASLD group. Additionally, the concentrations of HDL subclasses H1FC, H1PL, H1A1, H2FC, and H2A2 decreased in the disease group. Interestingly, although most HDL subclasses exhibited reduced concentrations in the disease group, the H4TG concentration increased. Furthermore, the differential lipoproteins displayed in both the lean and overweight groups exhibited a heightened diagnostic value only in the lean group.

**Figure 5:**
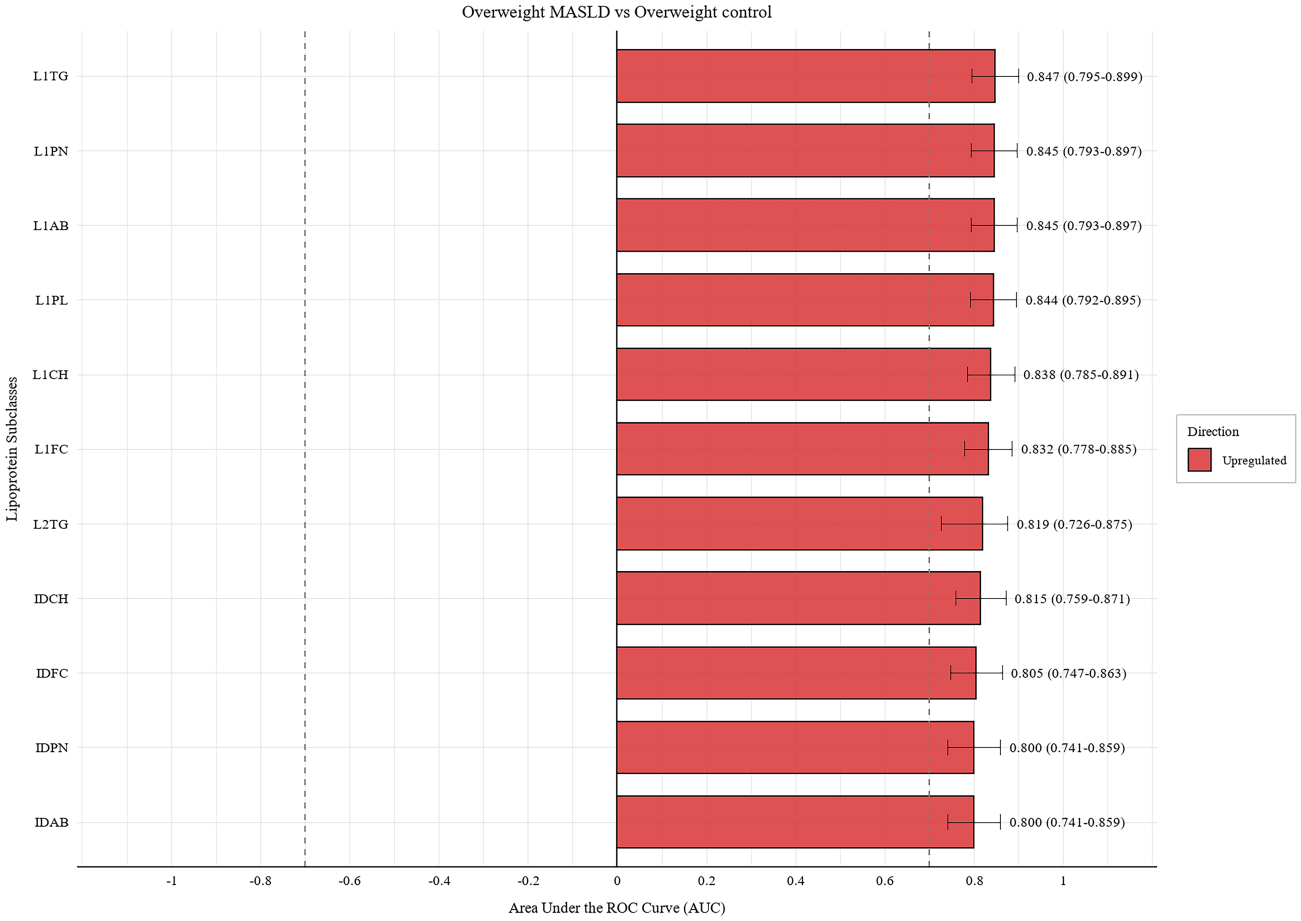
Diagnostic performance of candidate biomarkers with AUC > 0.8 in the overweight MASLD group vs. the overweight control group. Bar length represents the area under the curve (AUC) for each candidate lipoprotein subclass, with error bars indicating the 95% confidence interval. Lipoproteins highlighted in red and blue denote up- and down-regulated subclasses, respectively.

In the overweight group, we identified 37 lipoproteins with an AUC > 0.7 (Figure 5; Supplementary Figure 9), of which 11 demonstrated high diagnostic capability (AUC > 0.8) (Figure 5). Changes in the concentrations of lipoprotein subclasses in the disease group compared with those in the healthy group are shown in Supplementary Figure 4. Bootstrap analysis of 37 lipoproteins also showed that these lipoproteins also had high stability (Supplementary Figure 11). Upregulated lipoproteins unique to the overweight group included ABA1, LDTG, IDPL, L1PN, L1CH, L1FC, L1PL, L1AB, L2TG, and L3TG, whereas downregulated lipoproteins included L5CH, L5FC, L5PL, L6CH, L6FC, L6PL, and H3PL.

ROC curve analysis was similarly conducted for the overweight and lean MASLD groups, resulting in the identification of eight lipoprotein subclasses with good diagnostic performance (AUC > 0.7) (Supplementary Figure 5).

**Figure 6:**
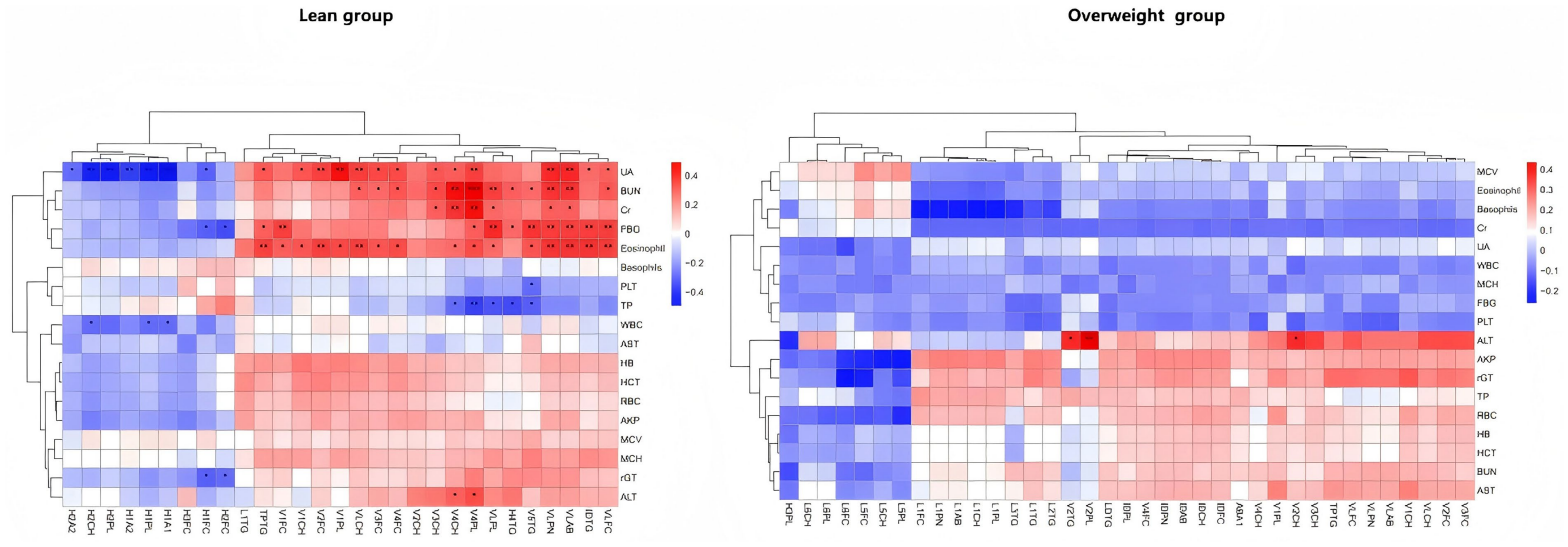
Correlation between lipoprotein level and clinical indexes. Pearson correlation heat map showing the correlation between clinical indexes and lipoprotein level in the lean group and the overweight group. Statistical significance is denoted by an asterisk (*p < 0.05, **p < 0.01, ***p < 0.001).

Significant differences in lipoprotein levels with respect to disease severity and obesity were supported by their correlations with complete blood counts as well as liver and kidney function test parameters (Figure 6; Supplementary Table 2). We selected lipoprotein subclasses with AUC > 0.7 and parameters exhibiting statistical differences in blood count and liver and kidney function tests for correlation analysis. The results indicated that in the lean group, HDL subclasses were negatively correlated with UA and fasting blood glucose (FBG), whereas VLDL subclasses were positively correlated with UA, blood urea nitrogen (BUN), Cr, FBG, eosinophils, and ALT, and negatively correlated with total protein (TP). In the overweight group, only the VLDL subclasses were positively correlated with ALT levels.

**Figure 7:**
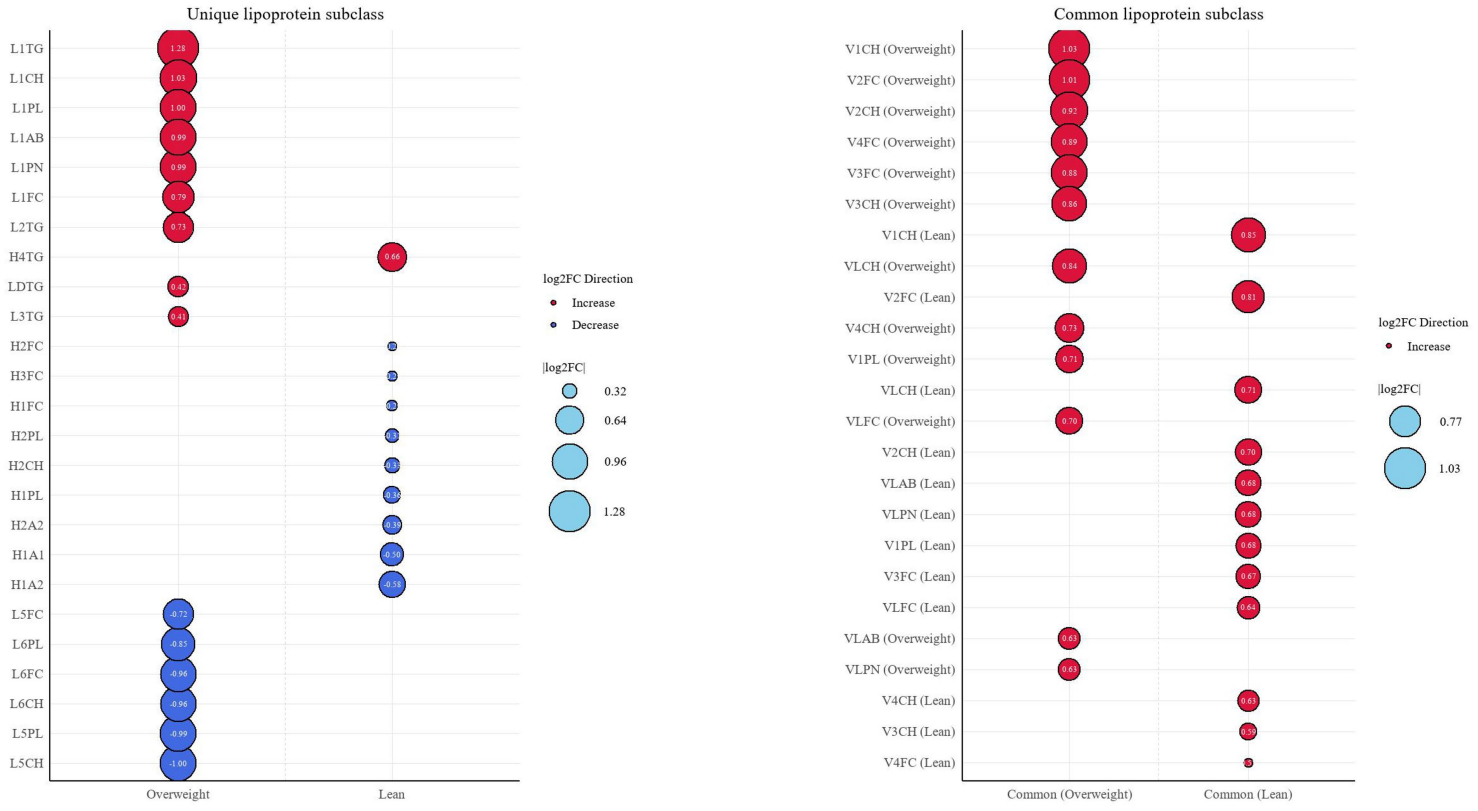
Summary of changes of serum lipoprotein profile in three groups. The red circle represents an increase, whereas the blue circle indicates a decrease.

**Figure 8:**
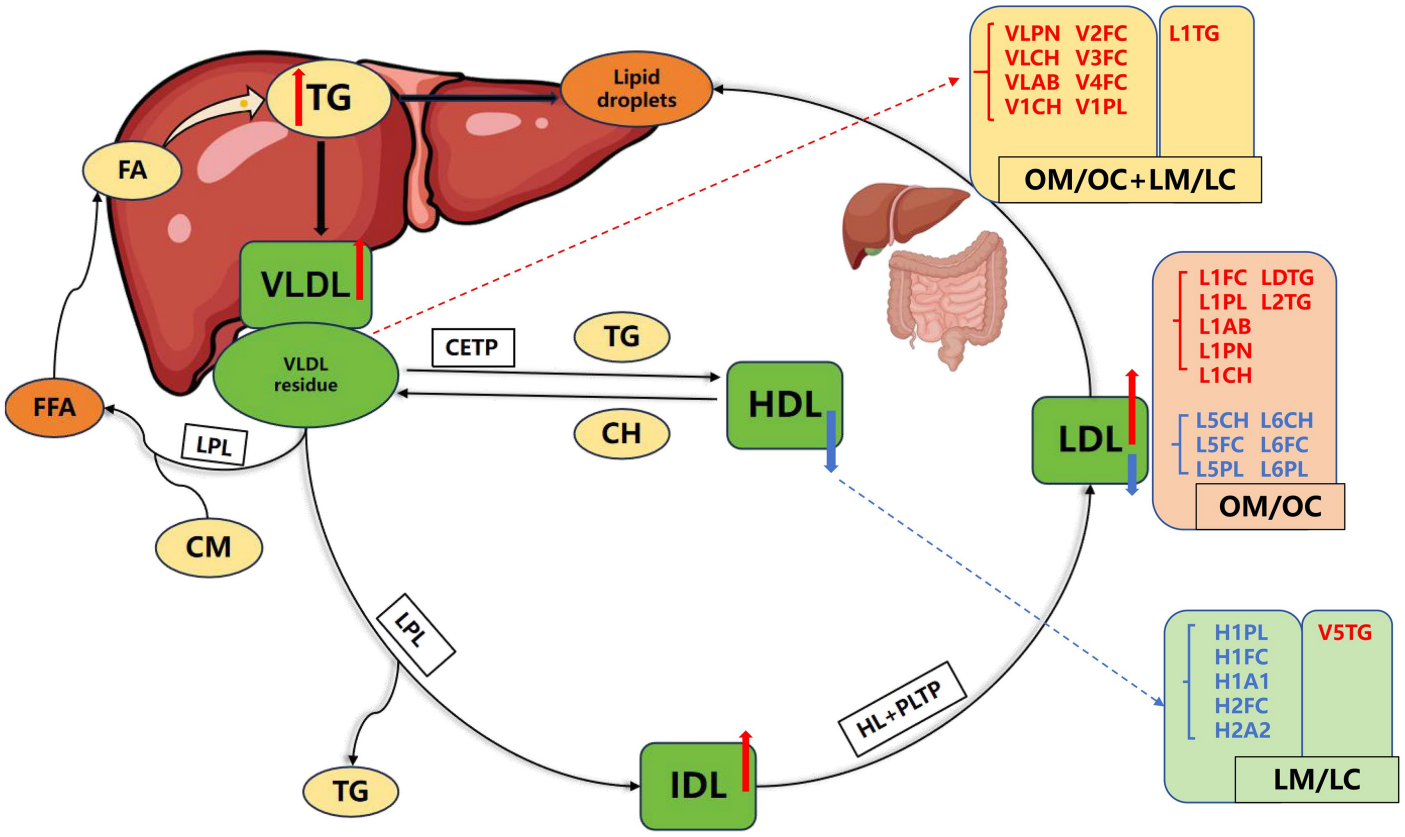
Schematic diagram of changes in serum lipoprotein profiles in three groups. The red arrows indicate an increase, and the blue arrows indicate a decrease.

We summarized the lipoproteins with a contribution score > 1 from the random forest analysis, as well as the lipoprotein subclasses with an AUC > 0.7 from the ROC analysis, and identified several specific lipoprotein subclasses that differentiated the lean group from the overweight group (Figures 7,8). Based on the uniqueness of these lipoproteins, we propose that lean and overweight patients with MASLD have different pathogenic mechanisms. The results revealed that lipoproteins in the lean MASLD group were primarily concentrated in the HDL and VLDL subclasses, whereas those in the overweight group were primarily comprised of LDL and VLDL. We hypothesized that the unique pathogenic mechanism of lean MASLD may be related to its difficulty in forming HDL-associated subclasses, whereas overweight MASLD may progress owing to the excessive generation of LDL subclasses. Moreover, the concentrations of LDL-5 and LDL-6 were reduced in the disease group, and their densities were similar to those of HDL, leading us to speculate that their functions may be similar to those of HDL subclasses. Our findings suggest significant differences in the lipoprotein composition between these groups, which may have important implications for diagnosis and therapy.

## 3 Discussion

MASLD is a metabolic disorder characterized by the abnormal deposition of fat in the liver and is closely associated with obesity (38). However, 10–20% of individuals with normal or low body fat percentages develop MASLD (39). Research has indicated that the occurrence of MASLD is not associated with BMI but rather with the pattern of fat deposition (40). Consequently, lean individuals are also at a risk of developing MASLD.

There is growing recognition that the pathogenesis of MASLD differs significantly among patients with varying BMI. Our findings suggest that overweight and lean MASLD represent two distinct diseases, each with a unique mechanism and a unique lipid profile. Overweight MASLD typically correlates with insulin resistance and dyslipidemia, as evidenced by elevated LDL and VLDL lipoproteins (41). In contrast, lean patients with MASLD, despite having a lower BMI, exhibited a distinct lipid profile, particularly in terms of the composition of HDL subclasses.

MASLD is identified by fat deposition, and an overabundance fat accumulation leads to increased VLDL secretion (42, 43). Disruption of VLDL secretion results in triglyceride accumulation and the buildup of lipotoxic lipids, contributing to lipid abnormalities (28). Dyslipidemia is considered a hallmark of MASLD-related lipid abnormalities and elevates the risk of cardiovascular diseases, including atherosclerosis and coronary artery disease. Furthermore, an abundance of research has demonstrated that MASLD patients exhibit elevated concentrations of TC, LDL-C, non-HDL cholesterol, TG, and APOB, while the concentrations of HDL-C and APOA1 are diminished (8, 11). These findings align with our results, which indicate that levels of triglycerides, cholesterol, phospholipids, and apolipoprotein B (APOB) are elevated in both the lean and overweight MASLD cohorts.

Clinical data indicated that LDL-C levels were significantly elevated in the disease group, whereas HDL-C levels were significantly decreased. Further breakdown of the lipoprotein subclasses suggested that an increase in LDL-C was primarily associated with elevated levels of L1CH and L1FC, whereas a decrease in HDL-C was mainly linked to a reduction in H2CH, H1FC, H2FC, and H3FC levels. Differences in lipoprotein levels related to disease severity indicate that specific lipoprotein subclasses may serve as biomarkers for MASLD progression. In the lean MASLD group, most HDL subclasses were downregulated, whereas H4TG levels were increased, suggesting a disturbance in lipid metabolism. Elevation of serum triglycerides is a well-known phenomenon in MASLD, and our findings support the notion that changes in HDL composition reflect potential metabolic dysregulation. In contrast, overweight patients with MASLD exhibited an increase in larger, less dense LDL particles along with a decrease in smaller lipoproteins. These results underscore the importance of the lipoprotein profile in understanding the systemic effects of MASLD and suggest that modulation of different lipoprotein ranges may be beneficial for the treatment of MASLD.

Studies have indicated that phosphatidylcholine (PC) concentrations are diminished in patients with MASLD and MASH, and supplementation with PC may serve as an adjunctive therapy to improve ALT levels in these individuals (44, 45). Furthermore, we observed significant differences in phospholipid subclasses within the lipoproteins, which were associated with a high diagnostic rate for the disease. These findings suggest that disturbances in lipid metabolism, particularly those involving phospholipids, are potential therapeutic targets for MASLD.

In this study, we observed elevated concentrations of a series of triglyceride-rich VLDL subclasses (e.g., VLFC, VLAB, VLPL, V1FC, V4PL, and V5TG) in lean MASLD patients, which showed significant positive correlations with blood glucose levels (Figure 6). This finding is highly consistent with the pathophysiological model of insulin resistance (31, 41). Specifically, the state of insulin resistance promotes lipolysis and reduces lipoprotein lipase activity, collectively leading to increased production and impaired clearance of VLDL and its remnants. This mechanism likely underlies the emergence of such an “atherogenic” lipoprotein profile in MASLD patients. It also partly explains the well-established association between MASLD, type 2 diabetes, and increased cardiometabolic risk (46–48).

In contrast to overweight patients, lean MASLD exhibits a distinct pattern of lipoprotein abnormalities characterized by alterations in HDL subclasses. We found that levels of specific, less-dense HDL subclasses (e.g., H1FC, H2FC) were significantly inversely correlated with blood glucose. This phenomenon can be explained by the direct impact of insulin resistance on HDL metabolism: hyperinsulinemia may reduce HDL biogenesis, potentially via pathways such as promoting ABCA1 degradation (41). Concurrently, elevated triglycerides may accelerate the remodeling and clearance of HDL by enhancing cholesteryl ester transfer protein (CETP) activity (49). Consequently, lipid abnormalities in lean MASLD patients may manifest earlier as metabolic disturbances in functionally relevant HDL subclasses. This suggests that detailed lipoprotein subclass analysis could be more sensitive than the conventional lipid panel in detecting underlying metabolic dysregulation in individuals with a normal BMI.

APOB serves as a vital structural protein implicated in all forms of atherogenic dyslipidemia and plays a crucial role in lipid transport. Studies have demonstrated that VLDL, IDL, and LDL particles contain one APOB molecule. Therefore, the concentration of APOB in the bloodstream is considered a marker for the quantity of circulating atherogenic lipoproteins, and APOB is regarded as a more accurate risk indicator than TC or LDL cholesterol (50). Using NMR spectroscopy, we found that the number of APOB particles, reflected by the VLDL concentration indicators VLPN and VLAB, was elevated in MASLD patients, further suggesting a heightened susceptibility to atherosclerosis in this population. Additionally, epidemiological research has indicated an association between serum APOB concentration and an elevated risk of MASLD (11). Conversely, APOA1 is an antiatherogenic lipoprotein primarily responsible for cholesterol in HDL. Our study revealed decreased H2CH and H1A1 levels, suggesting that MASLD increased the risk of atherosclerosis. Investigations have revealed that the mechanisms underlying disease progression may be related to ectopic deposition, lipid peroxidation, and triglyceride endocytosis (7).

Analysis of lipoprotein profiles indicated that lean MASLD is characterized by specific HDL subclasses, which could play an essential role in managing inflammation and lipid metabolism. In contrast, overweight MASLD is characterized by certain LDL subclasses associated with insulin resistance and degree of liver injury. This distinction not only highlights the complexity of these two forms of MASLD but also suggests that they may require different diagnostic and therapeutic strategies. Importantly, our findings support the hypothesis that HDL subclasses can serve as potential diagnostic biomarkers for lean MASLD, whereas specific LDL subclasses may provide valuable insights into the diagnosis and treatment of overweight MASLD. These biomarkers improve our understanding of the disease processes and facilitate the implementation of tailored treatments in distinct patient populations.

In the analysis of clinical data, our results showed that lean individuals with MASLD were younger than overweight individuals. Studies have reported that after menopause, the decline in estrogen and estradiol levels affects hepatic lipid metabolism, leading to hepatocyte injury and liver fibrosis (36). Our study also observed that lean patients with MASLD were predominantly female. Although all clinical indicators of the enrolled MASLD patients were within acceptable limits, liver enzyme levels, such as ALT and AST, were notably higher in the lean MASLD group than in the lean control group, and the lean MASLD group had high LDL-C, hypertriglyceridemia, and higher fasting blood glucose levels. This indicates that lean individuals with MASLD are at an increased risk for metabolic syndrome and exhibit compromised liver function. Lean individuals with MASLD demonstrate elevated levels of various hematological indices, including RBC, WBC, HGB, and HCT, and studies have shown that RBC, WBC, and HGB levels are linked to metabolic syndrome and insulin resistance (IR) (37, 38). Additionally, HGB level has been reported to predict MASLD in combination with triglyceride and ferritin levels (40). These findings were consistent with the results of the present study.

This study observed that patients with lean MASLD exhibited significantly elevated levels of UA, BUN and Cr, accompanied by increased VLDL subclasses and decreased HDL subclasses. These findings suggest a shared pathophysiological basis between mild renal impairment and disordered lipid metabolism. Hyperuricemia may further exacerbate insulin resistance and hepatic lipid accumulation by promoting oxidative stress and inflammatory responses (51). Moreover, alterations in white blood cell count and eosinophil ratio indicate that a subclinical inflammatory state may be associated with lipoprotein remodeling, particularly in lean MASLD. Future studies should further investigate the causal relationships between these clinical parameters and lipoprotein subclasses, as well as their synergistic roles in MASLD progression.

The correlation between lipoprotein subclass profiles and clinical parameters underscores their potential utility as diagnostic tools. Monitoring changes in specific lipoprotein subclasses may refine patient stratification in MASLD and inform individualized treatment strategies. By targeting distinct lipoprotein abnormalities, clinicians may improve therapeutic outcomes across different MASLD subtypes. Compared with existing non-invasive tools, the lipoprotein subclass markers proposed in this study demonstrate higher AUC values and may provide complementary pathophysiological information (52). Future research could explore integrating lipoprotein subclasses with current tools to establish a multidimensional, stage-specific risk assessment system for MASLD, thereby enhancing the accuracy of early identification and stratified management.

In this study, NMR-based metabolomic analysis of serum lipoprotein subclasses was performed in an Asian cohort with lean MASLD. Our results indicate that HDL subclasses represent promising candidate biomarkers for lean MASLD, whereas LDL subclasses may serve as distinctive biomarkers for overweight MASLD within the same population. These findings highlight phenotype-specific lipoprotein signatures in Asian patients with MASLD, supporting their potential utility in monitoring disease progression and as targets for intervention. Further investigation is warranted to elucidate the underlying mechanisms of these lipoprotein alterations and to assess the influence of modifiable lifestyle factors such as nutrition and exercise. Ultimately, therapeutic strategies aimed at modulating specific lipoprotein subclasses may offer promising avenues for improving MASLD outcomes.

## 4 Limitations of this study

The cross-sectional nature of this study precluded systematic collection of recent dietary and physical activity, all of which may influence lipoprotein metabolism. Although participants with diagnosed hyperlipidemia using lipid-lowering drugs were excluded, residual confounding from other lifestyle or pharmacological factors remains possible. Furthermore, the study was conducted in a single-center Asian population with a limited sample size and no external validation cohort, which may restrict the generalizability of the findings despite internal validation measures. Finally, as clinical data were anonymized and randomly sampled, individual-level integration of metabolomic and clinical variables was not feasible; therefore, the diagnostic utility of the identified lipoprotein biomarkers warrants validation in a prospective cohort with comprehensive covariate adjustment.

## 5 Methods

### 5.1 Study subjects

Participants were enrolled from Putuo Hospital, which is affiliated with Shanghai University of Traditional Chinese Medicine in China, from March 2019 to June 2024. In total, 2013 eligible subjects were included in this study. MASLD was diagnosed based on the presence of significant hepatic steatosis detected by abdominal ultrasound in the absence of other causes of fatty liver disease according to the “Guidelines for Prevention and Treatment of Metabolism related (Non alcoholic) Fatty Liver Disease (2024 Edition)” established by the National Seminar on Fatty Liver and Alcoholic Liver Disease of the Chinese Medical Association and the Expert Committee of Fatty Liver of the Chinese Medical Doctor Association (2024, China). To minimize subjective bias, each examination in this study was performed by two ultrasound physicians. The study protocol was approved by the Medical Ethics Committee, and all participants provided written informed consent.

#### 5.1.1 Inclusion criteria

This study will enroll participants aged 18 to 70 years. All participants must meet the diagnostic criteria for Metabolic Dysfunction-Associated Steatotic Liver Disease (MASLD). Diagnosis requires: (1) evidence of hepatic steatosis confirmed by non-invasive imaging, defined as either abdominal ultrasonography indicating “moderate or greater fatty liver” or a Controlled Attenuation Parameter (CAP) value ≥ 248 dB/m via transient elastography; and (2) absence of significant alcohol consumption (defined as ≤30 g/day for men and ≤20 g/day for women) for at least one year prior to enrollment.

Eligible participants will be stratified into two groups based on body mass index (BMI, using Asian criteria) and metabolic profile. The overweight/obese MASLD group is defined by a BMI ≥ 23 kg/m^2^. The lean MASLD group is defined by a BMI < 23 kg/m^2^ plus the presence of at least one additional criterion for metabolic dysfunction. These additional criteria include: central obesity (waist circumference ≥90 cm for men or ≥80 cm for women, Asian standards); elevated blood pressure or use of antihypertensive medication; dyslipidemia or use of lipid-lowering medication; dysglycemia (fasting plasma glucose ≥5.6 mmol/L, glycated hemoglobin A1c ≥ 5.7%), a diagnosis of type 2 diabetes mellitus, or use of anti-diabetic medication; or insulin resistance (Homeostatic Model Assessment of Insulin Resistance [HOMA-IR] index ≥2.5).

To ensure metabolic and biomarker stability, participants must have maintained a stable body weight (fluctuation <5%) and had no major changes to their medication regimen for metabolic conditions within the three months preceding enrollment. Written informed consent is required from all participants.

#### 5.1.2 Exclusion criteria

Individuals meeting any of the following criteria will be excluded to ensure cohort homogeneity and minimize confounding factors:

(1) Etiology of chronic liver disease other than MASLD, including viral hepatitis (positive for hepatitis B surface antigen or hepatitis C virus RNA), autoimmune liver disease, genetic hepatopathy (e.g., hemochromatosis, Wilson’s disease), or drug-induced liver injury.
(2) Evidence of advanced liver disease, such as clinical or imaging findings suggestive of cirrhosis, liver stiffness measurement ≥12.0 kPa, significant hepatic dysfunction (total bilirubin >1.5 times the upper limit of normal [ULN], international normalized ratio >1.3, or albumin <35 g/L), or suspected hepatocellular carcinoma.
(3) Uncontrolled major systemic diseases, including decompensated heart failure, end-stage renal disease, active malignancy, or active autoimmune disorders.
(4) Acute conditions within the past 4 weeks, such as infection, trauma, surgery, or requirement for antibiotic/anti-inflammatory therapy, which may influence systemic inflammation and biomarker levels.
(5) Chronic use of medications known to significantly affect hepatic metabolism or the biomarkers under investigation, including systemic corticosteroids, tamoxifen, amiodarone, and novel anti-diabetic/weight-loss agents (e.g., GLP-1 receptor agonists).
(6) History of bariatric surgery.
(7) Pregnancy or lactation.
(8) Any other condition deemed by the investigators to compromise participation or interfere with study objectives.

The overweight cohort included 732 and 353 individuals in the overweight disease and overweight control cohorts, respectively, whereas the lean cohort included 407 and 521 patients in the lean MASLD and lean control cohorts, respectively.

### 5.2 Clinical data collection

Venous blood samples were obtained following an overnight fast of no less than 12 h. The participants underwent a comprehensive physical examination, which included assessment of vital signs and anthropometric measurements. BMI was calculated by dividing the weight in kilograms by the square of the height in meters (kg/m^2^). Comprehensive medical history was collected using questionnaires. Laboratory tests included routine blood tests, fasting blood glucose levels, lipid profiles, biochemical measurements of liver and kidney function, and thyroid function tests.

### 5.3 Metabonomics analysis

The metabolomic sub-cohort was selected via stratified random sampling using SPSS (version 25). After stratifying the cohort by BMI and disease status, random numbers were generated within each stratum to draw proportionally balanced samples. Serum samples from 113 overweight MASLD patients, 107 overweight healthy individuals, 83 lean MASLD individuals, and 99 lean healthy controls were subsequently analyzed using metabolomic profiling performed in accordance with the Bruker IVDr standard operating procedure (SOP) (53). Serum samples were collected on a 600 MHz Avance NEO NMR spectrometer (Bruker Biospin GmbH, Rheinstetten, Germany) equipped with a BBI probe and a Samplejet autosampler with the temperature set at 4 °*C. prior* to sample collection, the instrument was first subjected to quality control experiments (B. I. BioBank QC™) in order to ensure consistent spectral quality. For each sample, solvent presaturation 1D 1H experiments were performed at 310 K using the NOESY pulse sequence, 32 scans were performed, totaling 98,304 data points covering a spectral width of 17,857 Hz. Corrections for baseline and phase were performed in Topspin 4.1.1 after data acquisition was completed.

### 5.4 Statistical analysis

Statistical analyses of clinical data were conducted using a two-tailed significance threshold of *p* < 0.05. Normality was evaluated using the Shapiro–Wilk test. Data that were normally or approximately normally distributed are expressed as mean ± standard deviation, while severely skewed distributions are reported as median (5th and 75th). For comparisons between multiple groups of non-normally distributed quantitative data, the Kruskal–Wallis H test was used, and the Bonferroni correction was applied to account for multiple comparisons of skewed data.

Before statistical analysis, metabolomic data were preprocessed to remove and impute missing values. Lipoprotein subclasses and metabolites with more than 30% of missing data were excluded. To address potential inter-batch variation during NMR detection and to ensure the effectiveness of our quality control (QC) protocol, we implemented a rigorous procedure. For each analytical batch, a QC experiment was first performed to verify instrument performance. Sample analysis proceeded only after the instrument passed this QC check. Furthermore, a detailed QC report was generated for every individual sample analyzed. Significant lipoprotein subcomponents and metabolites were identified by differential fold analysis, t-tests, and volcano plots. In all cases, statistical significance was set at *p* < 0.05. Considering the characteristics and nonlinear relationship of high-dimensional data, we use random forest as a tool for feature screening instead of building a prediction model. Random forest analysis was performed on the lipoprotein subcomponents data using “mean decrease Gini” as the metric to assess each variable’s contribution to each group, while the out-of-bag (OBB) error estimate was employed to assess model stability. A higher mean decrease in the Gini coefficient indicated a greater contribution of a feature to the classification accuracy of the model. Pearson’s correlation analysis was conducted to investigate the association between metabolites and clinical parameters. The area under the ROC curve (AUC) was calculated to assess the diagnostic efficacy of all the candidate biomarkers for MASLD. Bootstrap resampling stability analysis was utilized to assess the stability of the candidate biomarkers.

Data analysis and visualization were performed using SPSS v.26, R v.4.3.2, and GraphPad Prism v.9.0.

### 5.5 Sex as a biological variable

Our study examined both male and female participants, and sex was not considered a biological variable. These findings are not expected to be differentially relevant between males and females.

## Data Availability

The original contributions presented in the study are publicly available. This data can be found here: https://www.ebi.ac.uk/metabolights/MTBLS13821.

## Glossary

MASLD: Metabolic dysfunction-associated steatotic liver disease
HDL: High-density lipoprotein
LDL: Low-density lipoprotein
VLDL: Very low-density lipoprotein
IDL: Intermediate-density lipoprotein
BMI: Body mass index
FBG: Fasting blood glucose
WBC: White blood cell
RBC: Red blood cell
HGB: Hemoglobin
HCT: Hematocrit
MCV: Mean corpuscular volume
MCH: Mean corpuscular hemoglobin
MCHC: Mean corpuscular hemoglobin concentration
PLT: Blood platelets
RDW: Red blood cell distribution width
MPV: Mean platelet volume
BUN: Blood urea nitrogen
Cr: Creatinine
UA: Uric acid
GFR: Glomerular filtration rate
TB: Total bilirubin
DB: Direct bilirubin
AKP: Alkaline phosphatase
TP: Total protein
ALB: Albumin
*γ*-GT: γ-glutamyl transferase
CHE: Cholinesterase
ALT: Alanine aminotransferase
AST: Aspartate aminotransferase
TBA: Total bile acid
HDL-C: High-density lipoprotein cholesterol
LDL-C: Low-density lipoprotein cholesterol
TC: Total cholesterol
TG: Triglyceride
APOA1: Apolipoprotein A1
APOB: Apolipoprotein B
Lp(a): Lipoprotein(a)
TgAb: Thyroglobulin antibody
TPOAb: Thyroid peroxidase antibody
Tg: Thyroglobulin
TRAb: Thyrotropin receptor antibody
T3: Triiodothyronine
T4: Thyroxine
FT3: Free T3
FT4: Free T4
TSH: Thyroid stimulating hormone
CA-242: Carbohydrate antigen-242
CA50: Cancer antigen 50
CEA: Carcinoembryonic antigen
AFP: Alpha fetoprotein
CA19-9: Cancer antigen 19–9
CA72-4: Carbohydrate antigen 72–4
CA211: Cancer antigen 211
CA125: Cancer antigen 125
NSE: Neuron-specific enolase
SCC: Squamous cell carcinoma antigen
LPL: Lipoprotein lipase
LM: Lean MASLD
LC: Lean control
OM: Overweight MASLD
OC: Overweight control
FC: Fold change
