# Supplementary figures and images for: Dysregulated high-density lipoprotein and low-density lipoprotein subfractions increase metabolic dysfunction-associated steatotic liver disease risk: a study of patients across body mass index categories
Source: Front Nutr. 2026 Feb 13;13:1737860. doi: 10.3389/fnut.2026.1737860 (PMC12947705; doi:10.3389/fnut.2026.1737860)

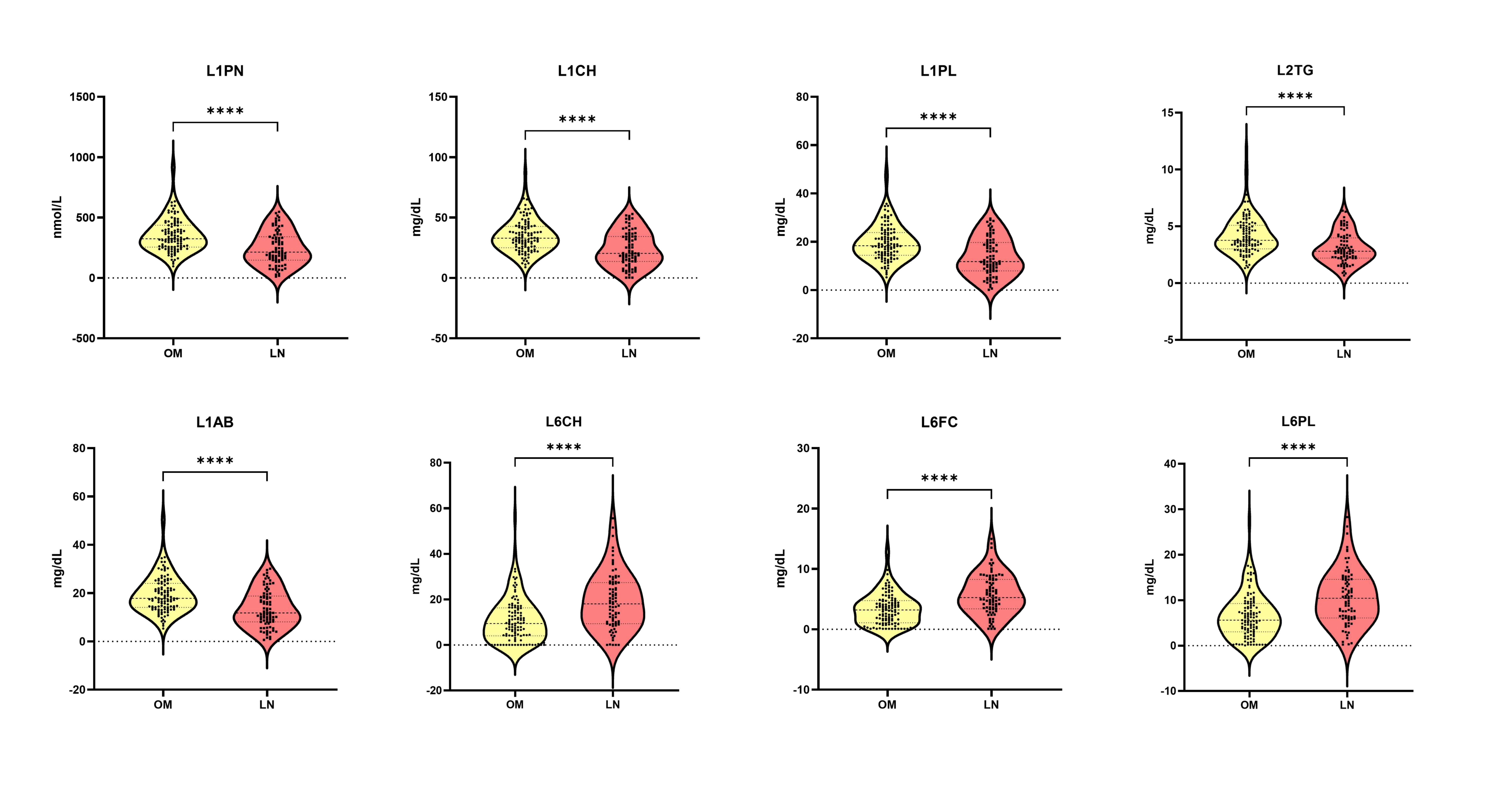

Supplement: Supplementary file 5 [file Image_5.PNG]

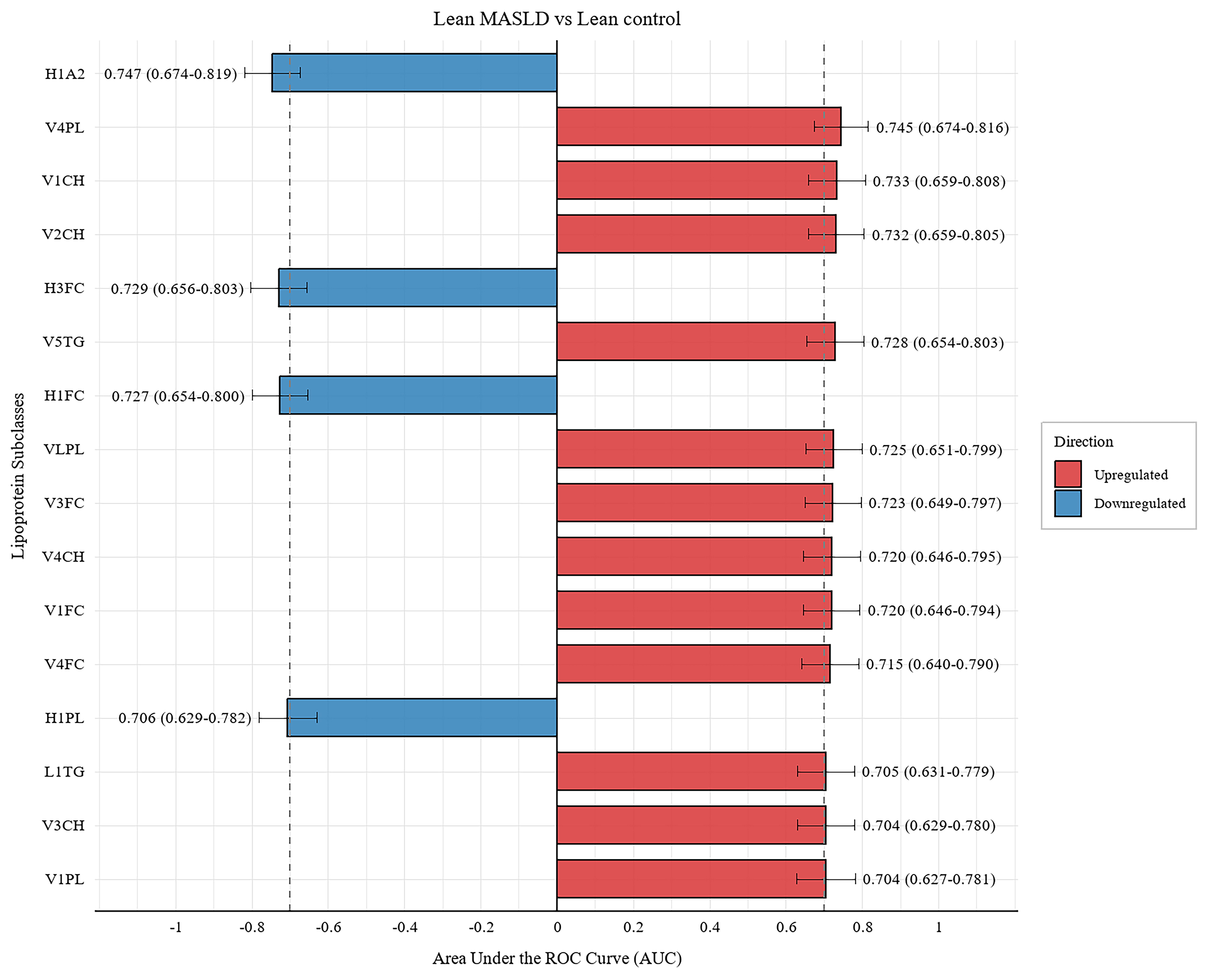

Supplement: Supplementary file 8 [file Image_8.PNG]

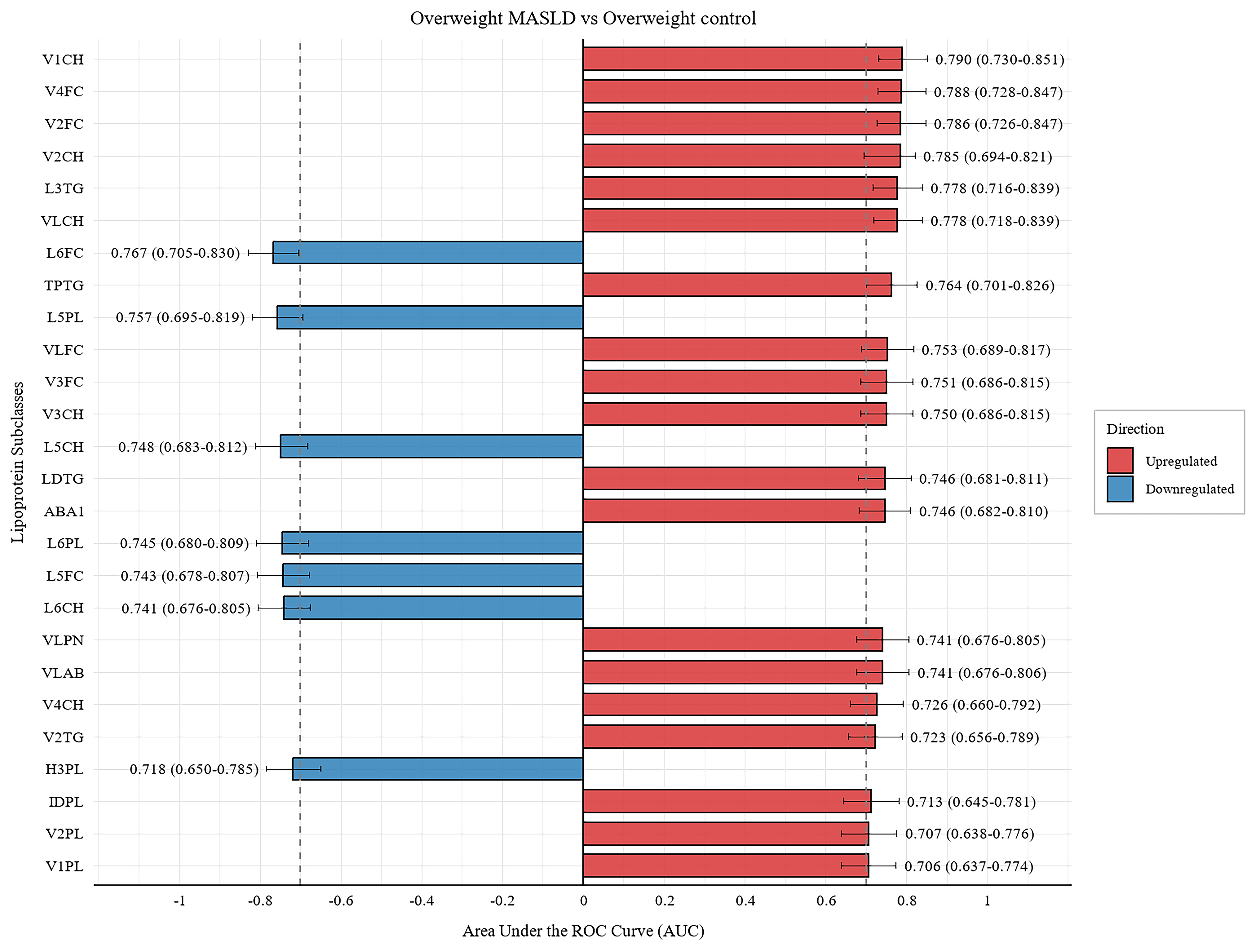

Supplement: Supplementary file 9 [file Image_9.PNG]
